# *De novo* Synthesis of SAA1 in the Placenta Participates in Parturition

**DOI:** 10.3389/fimmu.2020.01038

**Published:** 2020-06-09

**Authors:** Xiao-Wen Gan, Wang-Sheng Wang, Jiang-Wen Lu, Li-Jun Ling, Qiong Zhou, Hui-Juan Zhang, Hao Ying, Kang Sun

**Affiliations:** ^1^Center for Reproductive Medicine, Ren Ji Hospital, School of Medicine, Shanghai Jiao Tong University, Shanghai, China; ^2^Shanghai Key Laboratory for Assisted Reproduction and Reproductive Genetics, Shanghai, China; ^3^Shanghai First Maternity and Infant Hospital, School of Medicine, Tongji University, Shanghai, China; ^4^Department of Obstetrics and Gynecology, Ren Ji Hospital, School of Medicine, Shanghai Jiao Tong University, Shanghai, China; ^5^Shanghai International Maternal and Child Health Hospital, School of Medicine, Shanghai Jiao Tong University, Shanghai, China

**Keywords:** serum amyloid A, pregnancy, labor, preterm birth, inflammation

## Abstract

Serum amyloid A1 (SAA1) is an acute phase protein produced mainly by the liver to participate in immunomodulation in both sterile and non-sterile inflammation. However, non-hepatic tissues can also synthesize SAA1. It remains to be determined whether SAA1 synthesized locally in the placenta participates in parturition via eliciting inflammatory reactions. In this study, we investigated this issue by using human placenta and a mouse model. We found that SAA1 mRNA and protein were present in human placental villous trophoblasts, which was increased upon syncytialization as well as treatments with lipopolysaccharides (LPS), tumor necrosis factor-α (TNF-α), and cortisol. Moreover, significant increases in SAA1 abundance were observed in the placental tissue or in the maternal blood in spontaneous deliveries without infection at term and in preterm birth with histological chorioamnionitis. Serum amyloid A1 treatment significantly increased parturition-pertinent inflammatory gene expression including interleukin-1β (IL-1β), IL-8, TNF-α, and cyclooxygenase-2 (COX-2), along with increased PGF2α production in syncytiotrophoblasts. Mouse study showed that SAA1 was present in the placental junctional zone and yolk sac membrane, which was increased following intraperitoneal administration of LPS. Intraperitoneal injection of SAA1 not only induced preterm birth but also increased the abundance of IL-1β, TNF-α, and COX-2 in the mouse placenta. Conclusively, SAA1 can be synthesized in the human placenta, which is increased upon trophoblast syncytialization. Parturition is accompanied with increased SAA1 abundance in the placenta. Serum amyloid A1 may participate in parturition in the presence and absence of infection by inducing the expression of inflammatory genes in the placenta.

## Introduction

Inflammation of gestational tissues is implicated in the initiation of parturition at both term and preterm birth (1–4). Although infection-induced inflammation is an important cause of preterm delivery (2), non–infection-induced local immune remodeling in intrauterine tissues, also known as sterile inflammation, is an indispensable mechanism of labor onset at term as well (1, 4, 5). Moreover, sterile inflammation appears to be more frequent in gestational tissues in preterm labor with intact fetal membranes (6, 7). Therefore, understanding the mechanism of inflammation in parturition with or without infection may help develop strategies for prevention of preterm birth.

Serum amyloid A (SAA) belongs to acute phase proteins. Serum amyloid A family is consisted of multiple members including the most abundant serum amyloid A1 (SAA1) and other less prominent members such as SAA2α, SAA2β, SAA3, and SAA4 (8, 9). Serum amyloid A1 is massively secreted into the blood predominantly by the liver in the acute phase of responses to both sterile and non-sterile noxious stimuli (8, 9). Although the exact role of SAA1 in acute phase responses is not completely understood, accumulating evidence indicates that SAA1 may act as chemoattractant and immune modulator in either sterile and non-sterile inflammatory reactions via a number of receptors including RAGE (receptor for advanced glycation end products), TLR2 and TLR4 (toll-like receptor 2/4), P2X7 (P2X purinoceptor 7) receptor, and FPR2 (formyl peptide receptor 2) (8, 10–16).

In addition to the liver, SAA1 can also be synthesized in non-hepatic tissues (17). Our recent data have shown that SAA1 can be synthesized in human fetal membranes, which is significantly increased at parturition (18, 19). In the fetal membranes, SAA1 is not only involved in extracellular matrix remodeling in membrane rupture, but also stimulates the production of proinflammatory cytokines and prostaglandin E2 (PGE2), thereby participating in parturition (18–20). However, whether the placenta can also be a source of SAA1 in parturition is not known. Based on the current understanding of the role of SAA1 in sterile and non-sterile inflammation as well as the role of fetal membrane source of SAA1 in parturition (12, 16, 18–20), we hypothesized that SAA1 synthesized in the placenta may also take part in parturition by eliciting local pro-inflammatory responses in the presence or absence of infection. Here, we tested the hypothesis by using human placental tissue and trophoblasts as well as a mouse model.

## Materials and Methods

### Collection of Human Placenta Villous Tissue and Maternal Blood

Human placental villous tissue and maternal blood were obtained from pregnancies at term or preterm, with or without labor, in the presence or absence of infection with written informed consents under a protocol approved by the Ethics Committee of Ren Ji Hospital, School of Medicine, Shanghai Jiao Tong University. Women with gestational complications such as preeclampsia, fetal growth restriction, and gestational diabetes were excluded from the present study. Further information on recruited pregnant women is given in the following corresponding section.

### *In situ* Hybridization

To study the distribution of SAA1 mRNA in human placenta, *in situ* hybridization was performed on placental tissue obtained from uncomplicated term (38–40 weeks) pregnancies after elective cesarean section without labor [designated as term not in labor (TNL)]. The villous tissue was fixed with 4% paraformaldehyde in 1%0 diethyl pyrocarbonate (DEPC), and the paraffin-embedded tissue was sectioned at 4 μm in thickness for subsequent *in situ* hybridization using a customized kit containing digoxigenin-labeled antisense RNA probe against SAA1 mRNA (Boster, Wuhan, China). Briefly, after deparaffinization, the tissue section was digested with 3% proteinase K diluted in citric acid for 5 min at 37°C. After rinsing with phosphate-buffered solution (PBS), the section was post-fixed with 1% paraformaldehyde in 1%0 DEPC for 10 min at room temperature. Upon washing, the post-fixed section was incubated in hybridization solution containing the oligonucleotide probe at 37°C overnight. After incubation with blocking solution at 37°C for 30 min, the section was exposed to a biotin-conjugated anti-DIG antibody for 60 min at 37°C followed by incubation with streptavidin-biotin complex solution for 20 min at 37°C. Biotin–peroxidase and 3,3-diaminobenzidine were then added to develop a red brown color. For negative control, the section was incubated with a scrambled oligonucleotide probe. The slide was counterstained with hematoxylin and mounted for examination with a microscope (Carl Zeiss, Oberkochen, Germany).

### Immunohistochemical and Immunofluorescence Staining

To examine the distribution of SAA1 protein, placental villous tissue from TNL pregnancies was collected for immunohistochemical and immunofluorescent staining. Paraffin-embedded villous tissue was sectioned at 5 μm in thickness and was then deparaffinated. For immunohistochemical staining, endogenous peroxidase activity was quenched with 0.3% H_2_O_2_. After blocking, the section was incubated with a primary antibody against SAA1 (MAB30191; R&D System, Minneapolis, MN, USA) at 1:50 dilution or non-immune serum for negative control overnight at 4°C. After washing, the section was incubated with a secondary antibody conjugated with biotinylated horseradish peroxidase (HRP). The substrate 3-amino-9-ethyl carbazole (Vector Laboratories, Burlingame, CA, USA) was then added to develop peroxidase activity as a red color. The slide was counterstained with hematoxylin and mounted for examination under a microscope (Zeiss).

For immunofluorescent staining, the section was permeabilized with 0.4% Triton X-100 following deparaffination. After blocking, the section was incubated with primary antibodies against SAA1 (R&D System) at 1:50 dilution and 11β-hydroxysteroid dehydrogenase 2 (11β-HSD2) at 1:200 dilution (sc-20176; Santa Cruz Biotechnology, Dallas, TX, USA) overnight at 4°C followed by incubation with Alexa Fluor 488–labeled (green color) or Alexa Fluor 594–labeled (red color) secondary antibodies (Proteintech, Wuhan, China) against 11β-HSD2 and SAA1 primary antibodies, respectively, for 2 h. 11β-HSD2 is a well-described placenta glucocorticoid barrier and known to be present in the syncytiotrophoblast (21, 22), which was used as a marker for syncytiotrophoblast in this study. Nuclei were counterstained with DAPI (1 μg/mL, blue color). The slides were examined under a fluorescence microscope (Zeiss).

### Isolation and Culture of Human Placental Trophoblasts

Cytotrophoblast cells were isolated from TNL human placentas using a modified Kliman's method as described previously (23, 24). Briefly, after washing with ice-cold normal saline, placenta villous tissue was cut into small pieces and digested with 0.125% trypsin (Sigma Chemical Co., St. Louis, MO, USA) and 0.03% deoxyribonuclease I (Sigma) in Dulbecco modified eagle medium (DMEM) (Gibco, Grand Island, NY, USA) containing 1% antibiotics (Gibco). After digestion and centrifugation, isolated cytotrophoblasts were purified with a 5% to 65% Percoll gradient (GE Healthcare Bio-Sciences, Uppsala, Sweden), and cytotrophoblast cells between densities of 1.049 and 1.062 g/mL were collected for culture at 37°C in 5% CO_2_/95% air in DMEM containing 10% fetal calf serum (FCS) (Gibco) and 1% antibiotics to allow syncytialization *in vitro*. To visualize the status of syncytialization, hematoxylin-eosin staining was performed on cultured trophoblasts, which were examined using a microscope (Zeiss).

### Treatment of Cultured Human Placental Syncytiotrophoblasts

To observe changes of SAA1 expression before and after trophoblast syncytialization, isolated cytotrophoblasts were cultured for 3 and 48 h in the culture medium containing 10% FCS without any treatments. To examine whether cortisol, tumor necrosis factor-α (TNF-α), and lipopolysaccharides (LPS), the major product of gram-negative bacteria infection, could stimulate SAA1 production in syncytiotrophoblasts, syncytialized trophoblasts were treated with cortisol (1 μM; Sigma), TNF-α (10 ng/mL; Thermo Fisher Scientific, Carlsbad, CA, USA), and LPS (5 ng/mL; Sigma) in the absence of FCS for 24 h. RNA was then extracted from the cell for the measurement of SAA1 mRNA, and culture medium was collected for the measurement of SAA1.

To study the effects of SAA1 on interleukin-1β (IL-1β), interleukin-8 (IL-8), TNF-α, and cyclooxygenase-2 (COX-2) expression as well as PGE2 and PGF2α production, syncytiotrophoblasts were treated with recombinant human apo-SAA1 (10 ng/mL; PeproTech Inc., Rocky Hill, NJ, USA) for 24 h. To determine the involvement of TLR4 in the induction of IL-1β, IL-8, TNF-α, and COX-2 expression by SAA1, syncytiotrophoblasts were treated with SAA1 (10 ng/mL) in FCS-free medium in the presence or absence of a TLR4 specific inhibitor CLI095 (5 μM; Invitrogen, San Diego, CA, USA). To test whether the effect of SAA1 was ascribed to the trace amount of endotoxin contained in the recombinant human apo-SAA1 preparation, cells were treated with 1 pg/mL LPS, which is equivalent to the maximal amount of LPS contained in 10 ng of recombinant SAA1 according to the manual provided by the manufacturer. To further test whether the effect was ascribed to SAA1 *per se* or possible contaminating endotoxin, cells were treated with SAA1 (10 ng/mL) or LPS (5 ng/mL) in the presence or absence of an endotoxin inhibitor polymyxin B (10 μg/mL; Sigma) (25). RNA was extracted from above treated cells for measurements of IL-1β, IL-8, TNF-α, and COX-2 mRNA, and conditioned medium was collected for measurements of IL-1β, IL-8, TNF-α, PGE2, and PGF2α with enzyme-linked immunosorbent assay (ELISA) kits.

### Extraction of RNA and Analysis With Quantitative Reverse Transcription–Polymerase Chain Reaction

Extraction of total RNA from trophoblasts was conducted using a total RNA isolation kit (Foregene, Chengdu, China). After examination of RNA quality, mRNA was reverse transcribed to complementary DNA (cDNA) using a PrimeScript RT Master Mix Perfect Real Time Kit (Takara, Kyoto, Japan). The abundance of SAA1, IL-1β, IL-8, TNF-α, and COX-2, mRNA was determined with quantitative reverse transcription–polymerase chain reaction (qRT-PCR) using the above transcribed cDNA and power Sybr Premix Ex TaqTM (Takara) following a previously described protocol (26). GAPDH was used as an internal control for normalization. The relative mRNA abundance was quantified using the 2^−ΔΔ*Ct*^ method. The primer sequences used for qRT-PCR were as follows: GAPDH, 5′-CCCCTCTGCTGATGCCCCCA-3′ (forward) and 5′-TGACCTTGGCCAGGGGTGCT-3′ (reverse); SAA1, 5′-TTTCTGCTCCTTGGTCCTGG-′3 (forward) and 5′-CTCTGGCATCGCTGATCACT-3′ (reverse); IL-1β, 5′-CCACCTCCAGGGACAGGATA-3′ (forward) and 5′-AACACGCAGGACAGGTACAG-3′ (reverse); IL-8, 5′-TCTGTCTGGACCCCAAGGAA-3′ (forward) and 5′-ATGAATTCTCAGCCCTCTTCAA-3′ (reverse); TNF-α, 5-′CCCATGTTGTAGCAAACCCTC-3′ (forward) and 5′-TATCTCTCAGCTCCACGCCA-3′ (reverse); COX-2, 5′-TGTGCAA- CACTTGAGTGGCT-3′ (forward) and 5′-ACTTTCTGTACTGCGGGTG-3′ (reverse).

### Extraction of Cellular Protein and Analysis of COX-2 Protein Abundance With Western Blotting

Total cellular protein was extracted from treated syncytiotrophoblasts using an ice-cold RIPA lysis buffer (Active Motif, Carlsbad, CA, USA) containing a protease inhibitor cocktail (Roche, Basel, Switzerland) and a phosphatase inhibitor (Roche). Cellular COX-2 protein abundance was quantified following a standard Western blotting protocol. Briefly, 40 μg protein of each sample was electrophoresed in a 10% sodium dodecyl sulfate–polyacrylamide gel and transferred to a nitrocellulose blot membrane. After blocking, the blot was incubated with an antibody against COX-2 (cst-12282s; Cell Signaling Technology, Danvers, MA, USA) at 1:500 dilution overnight at 4°C. After washing, the membrane was incubated with a corresponding secondary antibody conjugated with HRP for 1 h. The bands with HRP peroxidase activity were detected using a chemiluminescence detection system (Millipore, Billerica, MA, USA) and visualized using a G-Box chemiluminescence image-capture system (Syngene, Frederick, MD, USA). Internal loading control was examined by probing the blot with a GAPDH antibody (60004-1; 1:10,000; Proteintech).

### Measurement of SAA1, IL-1β, IL-8, TNF-α, PGE2, and PGF2α Abundance With ELISA

Culture medium from treated trophoblasts was collected. Serum amyloid A1, IL-1β, IL-8, TNF-α, PGE2, and PGF2α concentrations were measured with ELISA kits (SAA1, IL-1β, IL-8, all from R&D System; TNF-α, Proteintech; PGE2 and PGF2α, Cayman Chemical Company, Ann Arbor, MI, USA) according to protocols provided by manufacturers.

To observe changes of SAA1 abundance in the placental tissue in labor, placentas were collected from spontaneous deliveries at term [designated as term with labor (TL)] as well as from TNL. Chunks of villous tissue were cut randomly from the maternal side of the placenta and then ground in liquid nitrogen. The ground tissue was homogenized and lysed in ice-cold RIPA lysis buffer containing the protease inhibitor cocktail. The supernatant was collected after centrifugation for the measurement of SAA1 with the ELISA kit. To observe changes of SAA1 levels in the maternal blood during spontaneous labor at term, maternal venous blood was collected from the same woman before (1–2 days before labor onset), after onset of labor and 24 h after delivery. The onset of labor was defined as regular uterine contractions (duration of 30 s with 5- to 6-min intervals) in the presence of full cervical dilation and descending of fetal presentation.

To examine changes of SAA1 levels in the maternal blood in preterm birth with or without infection, maternal blood was collected from infection-induced preterm labor and iatrogenic preterm birth without infection. The status of infection was determined by histological examination of neutrophil infiltration in the fetal membranes or placenta as well as positive bacteria culture with or without clinical signs of systemic infection. Iatrogenic preterm birth by cesarean section without infection and labor process was used as control for infection-induced preterm birth. Iatrogenic preterm birth includes pregnant women with placenta previa, scarred uterus, emergency fetal distress, and breech presentation with threatened preterm labor.

### Animal Study

C57BL/6 mice (Charles River, Beijing, China) were used in this study. Mice experimentation was conducted following accepted standards for animal care, which was approved by the Institutional Review Board of Ren Ji Hospital, School of Medicine, Shanghai Jiao Tong University. Mice aging from 10 to 13 weeks were mated. When a vaginal plug was present, it was counted as gestational day 0.5. In order to study the distribution of SAA1, mouse placenta and fetal membranes were collected and fixed on gestational day 18.5. Immunohistochemical staining of paraffin-embedded tissue sections was conducted following the same protocol as described above using a primary antibody against mouse SAA1 (AF2948, 1:100; R&D System). To study gestational changes of SAA1, mouse placenta and fetal membranes were collected on gestational days 16.5 and 18.5. Serum amyloid A abundance was determined with a mouse SAA ELISA kit (DY2948-05; R&D System), which measured both SAA1 and SAA2 according to instructions from the manufacturer. To compare amounts of SAA1 in the placenta and fetal membranes in infection-induced preterm birth, LPS [5 mg/kg body weight (BW)] or PBS was injected intraperitoneally on gestational day 16.5. Some of the mice were allowed to deliver spontaneously for observation of delivery time, and some of the mice were sacrificed 6 h after injection for collection of placenta and fetal membranes to examine the abundance of SAA1 in these tissues. To observe whether injection of SAA1 can induce preterm birth, recombinant SAA1 (8 μg/kg BW) or PBS or equivalent maximal amount of LPS (0.8 ng/kg BW) contained in the above administered SAA1 was injected intraperitoneally on gestational day 16.5. Some of the mice were allowed to deliver spontaneously for observation of delivery time and demised fetus, and some of the mice were sacrificed 6 h after injection for collection of placentae to examine the abundance of IL-1β, TNF-α, and COX-2. Collected tissues were frozen in liquid nitrogen, and protein was extracted for measurements of COX-2, IL-1β, and TNF-α abundance with Western blot or ELISA, respectively, as described above.

### Statistical Analysis

All data are expressed as mean ± standard error of the mean (SEM). The number for each experiment indicates repeated experiments using different placentas or animals. After examination of normal distribution, paired or unpaired Student *t*-test or Mann–Whitney *U*-test was used to compare two groups. One-way analysis of variance followed by Newman–Keuls multiple-comparisons test was performed when assessing the differences among multiple groups. Fisher exact tests were applied to compare the preterm birth rate in mice study. Significance was set at *P* < 0.05.

## Results

### SAA1 Expression in Human Placenta

**Figure 1:**
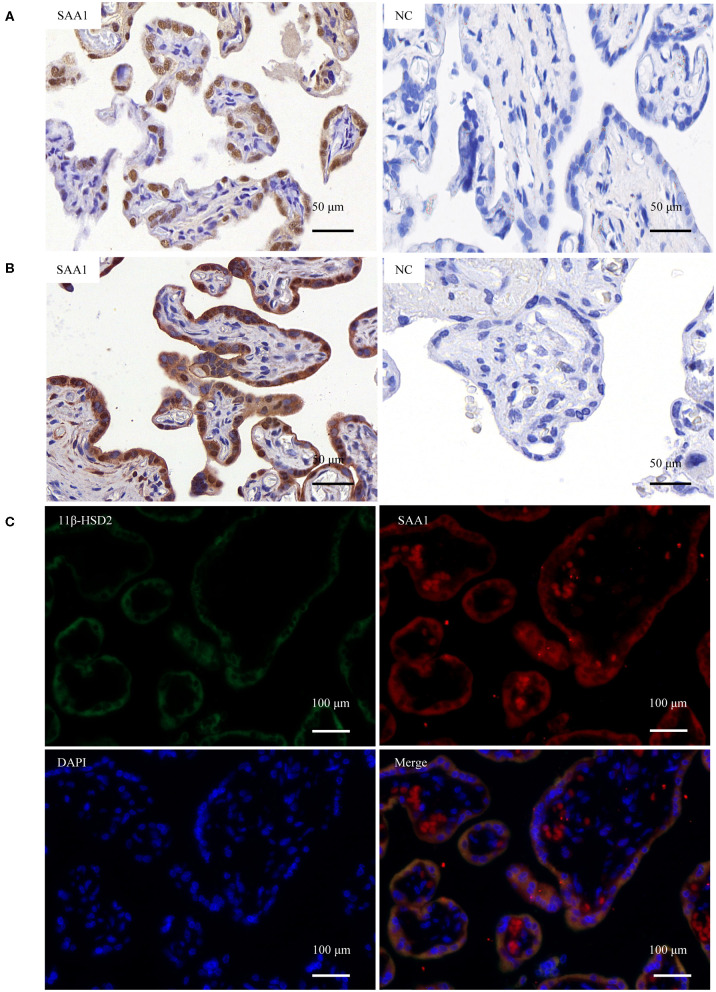
Expression of SAA1 in the human placenta at term. **(A)** *In situ* hybridization showed the presence of SAA1 mRNA (red brown) in the nuclei of the syncytial layer of the villous tissue. Scrambled RNA probe was used as the negative control (nc). **(B)** Immunohistochemical staining showed the presence of SAA1 (red) in the syncytial layer of the villous tissue. Non-immune serum was used as negative control (nc). **(C)** Immunofluorescence staining showed the colocalization of SAA1 (red) and 11β-HSD2 (green), a marker of syncytiotrophoblast, in the syncytial layer of the villous tissue. Nuclei (blue) were counterstained blue with DAPI.

**Figure 2:**
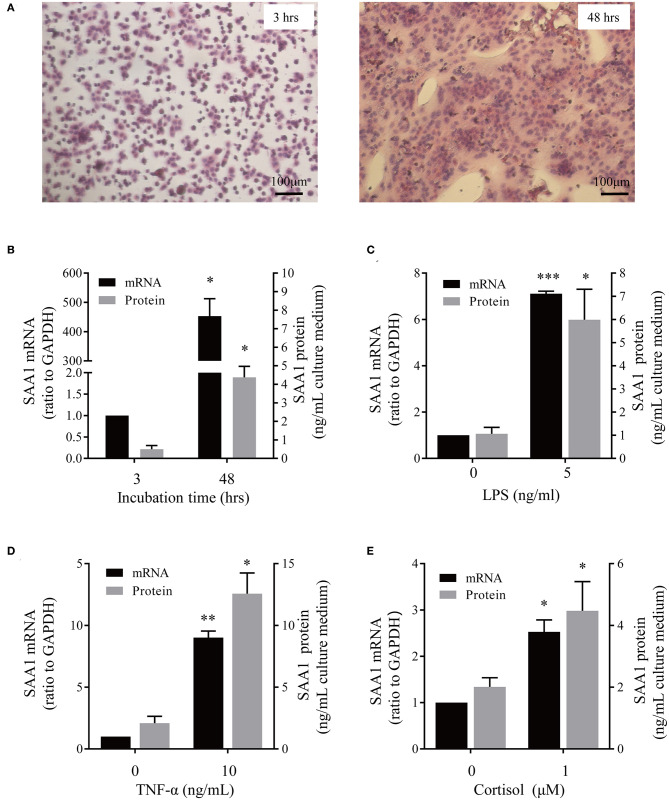
Effect of syncytialization and LPS, TNF-α and cortisol on SAA1 expression in human placental trophoblasts**. (A)** Hematoxylin-eosin staining of cultured trophoblasts showed cell fusion *in vitro*. **(B)** Changes of SAA1 mRNA and secretion before (3 h after cell plating) and after (48 h after cell plating) syncytialization (*n* = 3–4). **(C–E)** LPS (5 ng/mL, 24 h, **C**), TNF-α (10 ng/mL, 24 h; **D**), and cortisol (1 μM, 24 h; **E**) increased SAA1 mRNA and secretion in syncytiotrophoblasts (*n* = 4–5). Data are mean ± SEM. *P < 0.05, **P < 0.01, ***P < 0.001.

*In situ* hybridization revealed the presence of SAA1 mRNA mainly in the nuclei of the syncytial layer of placental villi (Figure 1A). Consistently, both immunohistochemical and immunofluorescent staining demonstrated that SAA1 protein distributed predominantly in the syncytial layer of placental villi, the identity of which was confirmed by staining for 11β-HSD2 (Figures 1B,C). Primary trophoblast cell culture study showed that the abundance of both SAA1 mRNA in trophoblasts and SAA1 protein in the culture medium was increased markedly during syncytialization (Figures 2A,B). LPS (5 ng/mL, 24 h), TNF-α (10 ng/mL, 24 h), and cortisol (1 μM, 24 h) treatments of syncytiotrophoblasts significantly increased SAA1 mRNA expression and secretion (Figures 2C–E).

### Changes of SAA1 in Human Placenta and Maternal Blood at Term and Preterm Parturition

**Figure 3:**
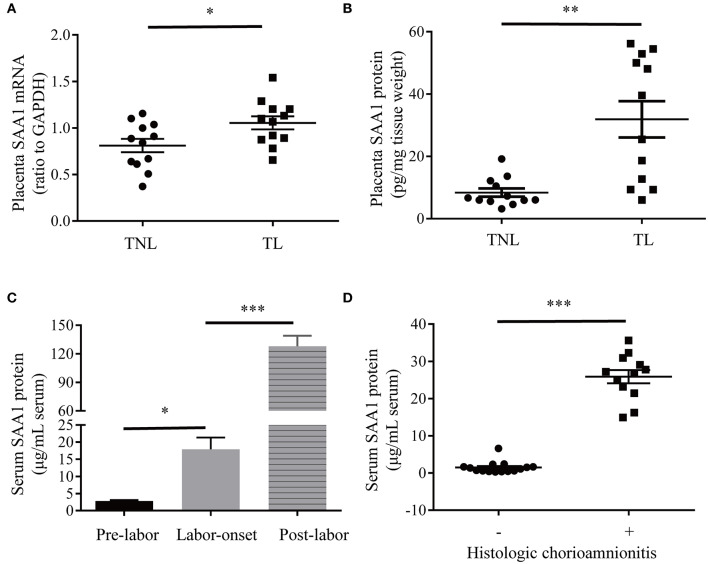
Increased SAA1 abundance in human placental tissue and maternal blood in labor at term and preterm. **(A,B)** Abundance of SAA1 mRNA and protein in human placenta tissue collected from elective cesarean section without labor at term (TNL) (*n* = 12) and spontaneous labor at term (TL) (*n* = 12). **(C)** Dynamic changes of serum SAA1 in pregnant women before, at the onset of, and 24 h after labor (*n* = 5). **(D)** Changes of SAA1 in maternal blood obtained from iatrogenic preterm birth without histologic chorioamnionitis (*n* = 15) and infection-induced preterm birth with histological chorioamnionitis (*n* = 12). Data are mean ± SEM. *P < 0.05, **P < 0.01, ***P < 0.001.

Demographic features of pregnant women of TNL and TL groups are illustrated in Table 1. There were no significant differences in gravity, parity, gestational age, maternal age, and fetal birth weight between TNL and TL groups. The abundance of SAA1 mRNA and protein was significantly increased in the placental villous tissue in TL group as compared with that in TNL group (Figures 3A,B). Moreover, longitudinal data of the same pregnant woman showed that the abundance of SAA1 in maternal blood was significantly increased at the onset of labor as compared with that of prelabor. Of interest, SAA1 abundance was further increased in maternal blood 24 h after labor (Figure 3C).

**Table 1:** Demographic features of term pregnant women (mean ± SEM).

**Demographic features**	**TNL (*n* = 12)**	**TL (*n* = 12)**	***P*-value**
Maternal age, years	30.92 ± 0.9	30.33 ± 0.71	0.61
Gravidity, median (range), *n*	2 (1–3)	2 (1–3)	0.42
Parity, median (range), *n*	1 (0–2)	1 (1–2)	0.13
Gestational age at delivery, weeks	38.9 ± 0.2	39.4 ± 0.2	0.07
Birth weight, g	3,219 ± 314.7	3,389 ± 120.1	0.78

As illustrated in Table 2, there are no significant differences in gravity, parity, gestational age, maternal age, and fetal birth weight between preterm birth group with infectious histological chorioamnionitis and iatrogenic preterm birth group without labor/histologic chorioamnionitis. A dramatic increase in SAA1 abundance was observed in maternal blood in preterm birth with infectious histologic chorioamnionitis as compared with that of iatrogenic preterm birth group (Figure 3D).

**Table 2:** Demographic features of pregnant women with preterm birth (mean ± SEM).

**Demographic features**	**Preterm birth without histological**	**Preterm birth with infectious histological**	***P*-value**
	**chorioamnionitis (*n* = 15)**	**chorioamnionitis (*n* = 12)**	
Maternal age, years	30 ± 1	31.5 ± 1	0.30
Gravidity, median (range), *n*	2 (1–4)	2 (1–5)	0.82
Parity, median (range), *n*	1 (0–1)	0 (0–2)	0.11
Gestational age at delivery, weeks	33.5 ± 0.7	33.1 ± 0.8	0.72
Birth weight, *g*	2,272 ± 133.9	2,104 ± 162.8	0.42

### Effect of SAA1 on the Expression of Genes Pertinent to Inflammation and Parturition in Human Placental Trophoblasts

**Figure 4:**
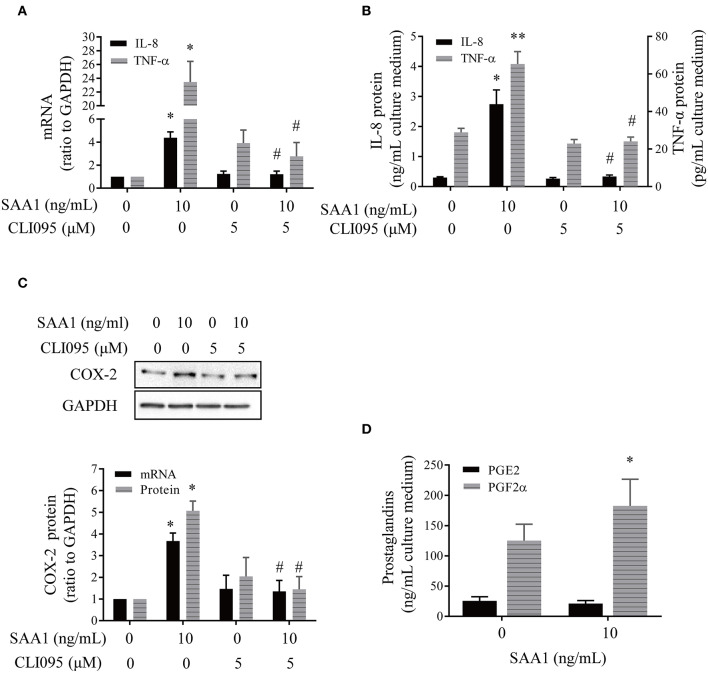
Effects of SAA1 on the expression of genes pertinent to parturition in human placental syncytiotrophoblasts. **(A,B)** Effects of SAA1 (10 ng/mL, 24 h) on IL-8, TNF-α mRNA, and secretion in the presence or absence of a TLR4 antagonist CLI095 (5 μM, 24 h) (*n* = 3–4). **(C)** Effects of SAA1 (10 ng/mL, 24 h) on COX-2 mRNA and protein in the presence or absence of a TLR4 antagonist CLI095 (5 μM, 24 h) (*n* = 3). Top panel is a representative immunoblot. **(D)** Effects of SAA1 (10 ng/mL, 24 h) on PGE2 and PGF2α secretions (*n* = 4). Data are mean ± SEM. *P < 0.05 vs. control (0); **P < 0.01 vs. control (0); ^#^*P* < 0.05 vs. SAA1.

To investigate whether SAA1 affects the expression of genes pertinent to inflammation and parturition, we treated syncytiotrophoblasts with recombinant SAA1 (10 ng/mL, 24 h) in the presence or absence of a TLR4 receptor antagonist CLI095 (5 μM). Serum amyloid A1 significantly increased the abundance of proinflammatory gene mRNA including IL-8 and TNF-α (Figure 4A). Concentrations of IL-8 and TNF-α in the culture medium were also significantly increased by SAA1 treatment (Figure 4B). Although IL-1β mRNA was increased in syncytiotrophoblasts by SAA1 treatment (Supplementary Figure 1), IL-1β was hardly detectable in the culture medium. Serum amyloid A1 treatment also increased COX-2 mRNA and protein abundance in syncytiotrophoblasts along with increased PGF2α abundance in the culture medium (Figures 4C,D). However, PGE2 level was much lower than PGF2α in the culture medium of syncytiotrophoblasts, which was not affected by SAA1. The effects of SAA1 on IL-1β (Supplementary Figure 1), IL-8, TNF-α, and COX-2 were blocked by CLI095 (Figures 4A–C).

The endotoxin inhibitor polymyxin B (10 μg/mL) could only block the induction of proinflammatory genes by endotoxin (5 ng/mL) but not by SAA1 (10 ng/mL) (Supplementary Figure 2A). Consistently, the trace amount of endotoxin (1 pg/mL), which is equivalent to the maximal amount of endotoxin contained in 10 ng/mL recombinant SAA1, failed to affect the expression of proinflammatory genes (Supplementary Figure 2B). These results suggest that the observed effects of SAA1 were specific but not due to the trace amount of endotoxin contained in recombinant SAA1.

### Expression of SAA1 in the Mouse Placenta and Fetal Membranes

**Figure 5:**
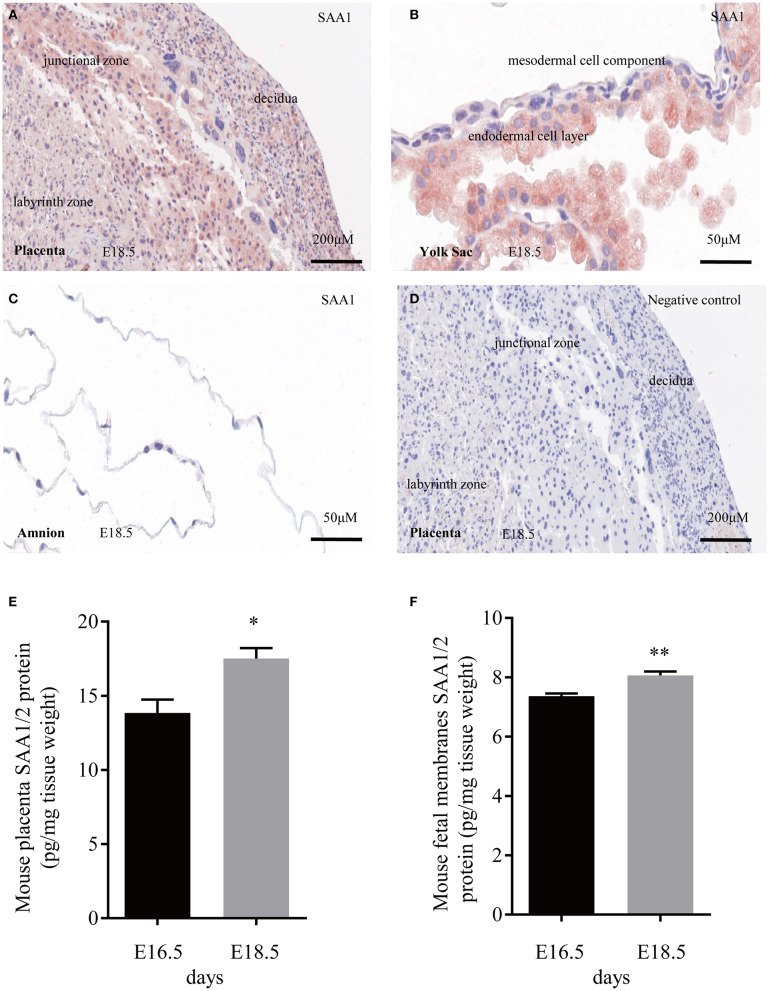
Expression of SAA1/2 in mouse placenta and fetal membranes. **(A–C)** Immunohistochemical staining showed the presence of SAA1 (red) in the junctional zone of the placenta **(A)** and the yolk sac membrane **(B)**, but not in the amnion **(C)** at gestational day 18.5. **(D)** negative control. **(E,F)** Increased SAA1/A2 abundance in mouse placenta and fetal membranes from gestational days 16.5 to 18.5 (*n* = 3). Data are mean ± SEM. *P < 0.05, **P < 0.01.

**Figure 6:**
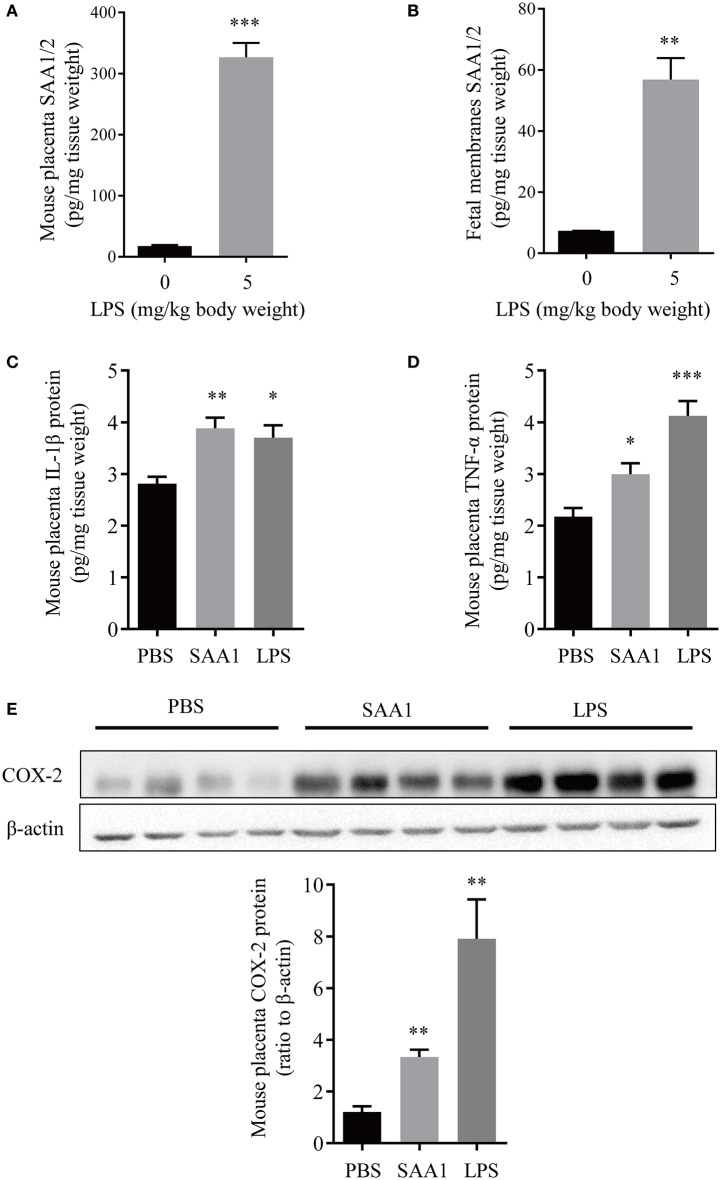
Induction of proinflammatory factors in placenta and fetal membranes by intraperitoneal administration of LPS or SAA1 in the mouse. **(A,B)** Induction of SAA1/2 expression in mouse placenta **(A)** and fetal membranes **(B)** by intraperitoneal administration of LPS (5 mg/kg BW, 6 h) at gestational day 16.5 (*n* = 3). **(C–E)** Induction of IL-1β **(C)**, TNF-α **(D)** and COX-2 **(E)** in the mouse placenta by intraperitoneal administration of LPS (5 mg/kg BW, 6 h) and SAA1 (8 μg/kg BW, 6 h) at gestational day 16.5 (*n* = 4). Data are mean ± SEM. *P < 0.05, **P < 0.01, ***P < 0.001.

Immunohistochemical staining of the mouse placenta and fetal membranes showed that SAA1 was present in the junctional zone of the placenta and the yolk sac membrane but not in the amnion of fetal membranes (Figures 5A–D). The abundance of SAA1/2 was significantly increased in mouse placenta and fetal membranes on gestational day 18.5 as compared with that on gestational day 16.5 (Figures 5E,F). Furthermore, SAA1/A2 abundance was significantly increased in placenta and fetal membranes by intraperitoneal injection of LPS (5 mg/kg BW) (Figures 6A,B). Intraperitoneal injection of SAA1 (8 μg/kg BW) on gestational day 16.5 induced preterm birth by 0.5 to 1 day with no demised fetus, and injections of PBS had no effect on delivery time (Table 3). All the three mice injected with equivalent maximal amount of LPS (0.8 ng/kg BW) contained in the injected recombinant SAA1 (8 μg/kg BW) delivered at term (19.5 days), suggesting that SAA1 *per se* rather than the trace amount of endotoxin contained in SAA1 preparation caused preterm birth. Moreover, intraperitoneal injection of SAA1 (8 μg/kg BW) or LPS (5 mg/kg BW) significantly increased the abundance of IL-1β, TNF-α, and COX-2 proteins in the mouse placenta (Figures 6C–E).

**Table 3:** Pregnancy outcome in mice administered with SAA1 and PBS.

**Treatments**	**Preterm birth dam/total number of**	**Fetal survival rate**
	**dam (preterm birth rate, %)**	**per dam (%)**
SAA1 (8 μg/kg BW)	5/7 (71.4%)^*^	100%
PBS (200 μL)	0/5 (0%)	100%

*BW, body weight*.

* *P < 0.05 against with PBS*.

## Discussion

It is widely accepted that the liver is the major source of drastically increased SAA1 in the blood during the acute phase of responses (8, 15, 27). However, non-hepatic tissues including fetal membranes can also synthesize SAA1 (17, 18). Previous studies have demonstrated the presence of SAA transcripts in placental trophoblast cell lines and possibly its protein in placental villous trophoblasts (28, 29). In the present study, we provided evidence that SAA1 could indeed be synthesized in the human placenta because both SAA1 mRNA and protein were present in placenta villous trophoblasts. Given the distribution of SAA1 was mainly in the syncytial layer of the villous tissue and the expression of SAA1 increased significantly during trophoblast syncytialization, we believe that SAA1 is a major product of the syncytial layer of placental villi. Detection of SAA1 in the culture medium of trophoblasts indicates that placenta trophoblasts can secret SAA1, suggesting that the fetal placenta may be an additional source of SAA1 in maternal blood in addition to maternal liver in pregnancy.

Inflammation of intrauterine tissues at parturition can be either sterile or infectious, depending on the presence of microbes or not (1–4). The evidence for the involvement of SAA1 in infection-induced inflammation is overwhelming. Here, we also found dramatic induction of SAA1 expression by LPS in the placenta. Additionally, pronounced increases in SAA1 levels in the maternal blood in preterm deliveries with infectious histologic chorioamnionitis as revealed in this study as well as in previous studies (30, 31) lend further support for the participation of SAA1 in infection-induced inflammation in labor process. What's more, the findings of stimulation of SAA1 expression and secretion by LPS in trophoblasts suggest that fetal placenta may contribute to the SAA1 pool together with maternal liver in the maternal blood in preterm birth with infection. However, we do not know how big the contribution from fetus or mother is at present.

In contrast to infection-induced inflammation, the triggers for sterile inflammation in labor process are not very well-resolved. Our findings that SAA1 abundance was increased in both maternal blood and placenta in spontaneous labor at term without infection strongly suggest participation of SAA1 in sterile inflammation of labor process as well, although cell aging and high mobility group box (HMGB) are also considered as triggers (6, 32, 33). Serum amyloid A1 may elicit sterile inflammation either directly on its own or indirectly through other sterile inflammation inducers such as HMGB1. It has been reported that SAA1 can stimulate HMGB1 release in macrophages (14). Our previous studies have shown that cortisol regenerated locally may be one of the upstream signals that stimulate SAA1 expression in the fetal membranes in sterile inflammation (34). Here, we demonstrated that cortisol, an important steroid hormone in parturition (35, 36), is an upstream inducer of SAA1 expression in the placenta as well. In addition to LPS and cortisol, proinflammatory cytokines such as TNF-α was also capable of stimulating SAA1 expression in the placenta. Because increased proinflammatory cytokine production may be present in intrauterine milieus in both infection- and non–infection-induced labor, we suppose proinflammatory cytokines are the upstream inducers of SAA1 expression in the placenta in both sterile and infectious labor process.

Studies in trophoblast-derived cell lines and extravillous trophoblasts indicate a role of SAA in trophoblast invasion but not in syncytialization (28, 29). Here, our findings that SAA1 induced the expression of a number of inflammatory factors including IL-1β, IL-8, TNF-α, and PGF2α are an indicator of involvement of SAA1 in inflammatory reactions in the placenta, which is in line with its inflammatory actions in fetal membranes. Because inflammation with increased production of proinflammatory cytokines and PGE2/PGF2α is an indispensable process of labor with or without infection (1–4), we trust that these proinflammatory actions of SAA1 in placenta and fetal membranes are an important route of SAA1 in the induction of labor in the presence or absence of infection in addition to its extracellular matrix remodeling effects in fetal membranes (18–20).

Previous studies have shown that there is increased TLR4 expression in the fetal membranes in spontaneous labor at both term and preterm regardless of infection (37), suggesting an important role of TLR4 in parturition. Of interest, mice with a spontaneous mutation for TLR4 gene are less likely to deliver at preterm after intrauterine inoculation of heat-killed bacteria or LPS than wild type (38, 39), further suggesting a crucial role of TLR4 in parturition. Here, we found that effects of SAA1 on proinflammatory gene expression could be abolished by a TLR4 antagonist in placental trophoblasts, which is consistent with the situation in fetal membranes (18), indicating that TLR4 mediates, at least in part, the proinflammatory actions of SAA1 in intrauterine tissues in parturition. As TLR4 is a receptor for many damage-associated molecular patterns (DAMPs) including SAA1 and HMGB1, how these DAMPs manage to exert their specific actions in the placenta or fetal membranes remains to be determined. It is possibly related to their differential affinities for TLR4 or the presence of other receptors that can bind DAMPs differentially.

Both PGE2 and PGF2α are crucial in labor induction with potent actions on myometrial contraction and cervical ripening. Of interest, it is believed that PGE2 is mainly produced by the amnion (40), whereas PGF2α is predominantly produced by the decidua and myometrium (40, 41). Here, we found that human placental syncytiotrophoblasts produced mainly PGF2α rather than PGE2, and PGF2α was inducible by SAA1. In addition, we found that IL-1β was not a major product of syncytiotrophoblasts either, although IL-8 and TNF-α were produced substantially.

It is noteworthy that SAA1 levels in the maternal blood were further increased after delivery. A previous study also found maximal levels of SAA in the maternal blood after delivery along with another acute phase protein C-reactive protein (42). Because these increases are observed after labor, we believe these increases are due to increased maternal SAA1 production. Because acute phase proteins are responsive to injury (8, 27), we speculate that these further increases after deliveries are a response to labor trauma and may be beneficial for tissue remodeling during trauma remedy.

In summary, we have demonstrated in this study that placental trophoblasts are capable of *de novo* synthesis of SAA1, which is increased during syncytialization and by LPS, TNF-α and cortisol stimulation. Serum amyloid A1 released by the placenta may participate in the onset of labor in the presence or absence of infection by stimulating the expression of parturition-pertinent inflammatory factors with consequently increased production of proinflammatory cytokines and PGF2α in the placenta.

## Data Availability Statement

The datasets generated for this study are available on request to the corresponding author.

## Ethics Statement

The studies involving human participants were reviewed and approved by The Ethics Committee of Ren Ji Hospital, School of Medicine, Shanghai Jiao Tong University. The patients/participants provided their written informed consent to participate in this study. The animal study was reviewed and approved by The Institutional Review Board of Ren Ji Hospital, School of Medicine, Shanghai Jiao Tong University.

## Author Contributions

X-WG, W-SW, and J-WL: performance of experiments, analysis and assembly of data. L-JL, QZ, H-JZ, and HY: analysis and interpretation of clinical data and collection of clinical samples. KS, W-SW, and X-WG: project design and management, planning experiment, analysis and assembly of data, manuscript writing.

## Conflict of Interest

The authors declare that the research was conducted in the absence of any commercial or financial relationships that could be construed as a potential conflict of interest.

## Supplementary Material

Supplementary Figure 1 Induction of IL-1β mRNA in human placental syncytiotrophoblasts by SAA1 (10 ng/mL, 24 h), which was blocked by CLI095 (5 μM, 24 h), a TLR4 inhibitor. *n* = 3. ^*^*P* < 0.05 vs. control (0); ^#^*P* < 0.05 vs. SAA1.

Supplementary Figure 2 Specificity examination of SAA1 effects in human syncytiotrophoblasts. **(A)** Polymyxin B (10 μg/mL, 24 h), an endotoxin inhibitor, had no effect on SAA1 (10 ng/mL)-induced changes in IL-1β, IL-8, TNF-α and COX-2 mRNA but blocked the effect of LPS (5 ng/mL) (*n* = 4). **(B)** Trace amount of LPS (1 pg/mL, 24 h), which is equivalent to the maximal amount contained in the recombinant SAA1, had no effect on the expression of IL-1β, IL-8, TNF-α, and COX-2 mRNA (*n* = 3). Data are mean ± SEM. ^*^*P* < 0.05, ^**^*P* < 0.01 vs. control (0); ^#^*P* < 0.05 vs. LPS.

## References

[1] Romero R Espinoza J Goncalves LF Kusanovic JP Friel LA Nien JK. Inflammation in preterm and term labour and delivery. Semin Fetal Neonatal Med. (2006) 11:317–26. 10.1016/j.siny.2006.05.001 16839830 PMC8315239

[2] Romero R Espinoza J Kusanovic JP Gotsch F Hassan S Erez O The preterm parturition syndrome. BJOG. (2006) 113 (Suppl. 3):17–42. 10.1111/j.1471-0528.2006.01120.x PMC7062298 17206962

[3] Challis JR Lockwood CJ Myatt L Norman JE Strauss JF 3rd, Petraglia F. Inflammation and pregnancy. Reprod Sci. (2009) 16:206–15. 10.1177/1933719108329095 19208789

[4] Bukowski R Sadovsky Y Goodarzi H Zhang H Biggio JR Varner M. Onset of human preterm and term birth is related to unique inflammatory transcriptome profiles at the maternal fetal interface. PeerJ. (2017) 5:e3685. 10.7717/peerj.3685 28879060 PMC5582610

[5] Keelan JA Blumenstein M Helliwell RJ Sato TA Marvin KW Mitchell MD. Cytokines, prostaglandins and parturition–a review. Placenta. (2003) 24 (Suppl. A):S33–46. 10.1053/plac.2002.0948 12842412

[6] Romero R Miranda J Chaiworapongsa T Korzeniewski SJ Chaemsaithong P Gotsch F. Prevalence and clinical significance of sterile intra-amniotic inflammation in patients with preterm labor and intact membranes. Am J Reprod Immunol. (2014) 72:458–74. 10.1111/aji.12296 25078709 PMC4192099

[7] Marcellin L Schmitz T Messaoudene M Chader D Parizot C Jacques S. Immune modifications in fetal membranes overlying the cervix precede parturition in humans. J Immunol. (2017) 198:1345–56. 10.4049/jimmunol.1601482 28031337

[8] Sun L Ye RD. Serum amyloid A1: structure, function and gene polymorphism. Gene. (2016) 583:48–57. 10.1016/j.gene.2016.02.044 26945629 PMC5683722

[9] Zhu S Wang Y Chen W Li W Wang A Wong S. High-Density Lipoprotein (HDL) Counter-Regulates Serum Amyloid A (SAA)-Induced sPLA2-IIE and sPLA2-V expression in macrophages. PLoS ONE. (2016) 11:e0167468. 10.1371/journal.pone.0167468 27898742 PMC5127586

[10] Sandri S Rodriguez D Gomes E Monteiro HP Russo M Campa A. Is serum amyloid a an endogenous TLR4 agonist? J Leukoc Biol. (2008) 83:1174–80. 10.1189/jlb.0407203 18252871

[11] Baranova IN Bocharov AV Vishnyakova TG Kurlander R Chen Z Fu D. CD36 is a novel serum amyloid A (SAA) receptor mediating SAA binding and SAA-induced signaling in human and rodent cells. J Biol Chem. (2010) 285:8492–506. 10.1074/jbc.M109.007526 20075072 PMC2832998

[12] Chen M Zhou H Cheng N Qian F Ye RD. Serum amyloid A1 isoforms display different efficacy at Toll-like receptor 2 and formyl peptide receptor 2. Immunobiology. (2014) 219:916–23. 10.1016/j.imbio.2014.08.002 25154907 PMC4252704

[13] Ebert R Benisch P Krug M Zeck S Meissner-Weigl J Steinert A. Acute phase serum amyloid A induces proinflammatory cytokines and mineralization via toll-like receptor 4 in mesenchymal stem cells. Stem Cell Res. (2015) 15:231–9. 10.1016/j.scr.2015.06.008 26135899

[14] Li W Zhu S Li J D'Amore J D'Angelo J Yang H. Serum amyloid a stimulates PKR expression and HMGB1 release possibly through TLR4/RAGE receptors. Mol Med. (2015) 21:515–25. 10.2119/molmed.2015.00109 26052716 PMC4607615

[15] Ye RD Sun L. Emerging functions of serum amyloid a in inflammation. J Leukoc Biol. (2015) 98:923–9. 10.1189/jlb.3VMR0315-080R 26130702 PMC6608020

[16] Baumann R Gube M Markert A Davatgarbenam S Kossack V Gerhards B Systemic serum amyloid A as a biomarker for exposure to zinc and/or copper-containing metal fumes. J Expo Sci Environ Epidemiol. (2018) 28:84–91. 10.1038/jes.2016.86 28176762

[17] Urieli-Shoval S Cohen P Eisenberg S Matzner Y. Widespread expression of serum amyloid A in histologically normal human tissues. Predominant localization to the epithelium J Histochem Cytochem. (1998) 46:1377–84. 10.1177/002215549804601206 9815279

[18] Li W Wang W Zuo R Liu C Shu Q Ying H. Induction of pro-inflammatory genes by serum amyloid A1 in human amnion fibroblasts. Sci Rep. (2017) 7:693. 10.1038/s41598-017-00782-9 28386088 PMC5429602

[19] Wang WS Li WJ Wang YW Wang LY Mi YB Lu JW. Involvement of serum amyloid A1 in the rupture of fetal membranes through induction of collagen I degradation. Clin Sci (Lond). (2019) 133:515–30. 10.1042/CS20180950 30683734

[20] Wang YW Wang WS Wang LY Bao YR Lu JW Lu Y. Extracellular matrix remodeling effects of serum amyloid A1 in the human amnion: implications for fetal membrane rupture. Am J Reprod Immunol. (2019) 81:e13073. 10.1111/aji.13073 30461130

[21] Zuo R Liu X Wang W Li W Ying H Sun K. A repressive role of enhancer of zeste homolog 2 in 11beta-hydroxysteroid dehydrogenase type 2 expression in the human placenta. J Biol Chem. (2017) 292:7578–87. 10.1074/jbc.M116.765800 28302719 PMC5418055

[22] Zhu P Wang W Zuo R Sun K. Mechanisms for establishment of the placental glucocorticoid barrier, a guard for life. Cell Mol Life Sci. (2019) 76:13–26. 10.1007/s00018-018-2918-5 30225585 PMC11105584

[23] Kliman HJ Nestler JE Sermasi E Sanger JM Strauss JF. 3rd. Purification, characterization, and *in vitro* differentiation of cytotrophoblasts from human term placentae. Endocrinology. (1986) 118:1567–82. 10.1210/endo-118-4-1567 3512258

[24] Wang W Li J Ge Y Li W Shu Q Guan H. Cortisol induces aromatase expression in human placental syncytiotrophoblasts through the cAMP/Sp1 pathway. Endocrinology. (2012) 153:2012–22. 10.1210/en.2011-1626 22315456

[25] Cardoso LS Araujo MI Goes AM Pacifico LG Oliveira RR Oliveira SC. Polymyxin B as inhibitor of LPS contamination of Schistosoma mansoni recombinant proteins in human cytokine analysis. Microb Cell Fact. (2007) 6:1. 10.1186/1475-2859-6-1 17201926 PMC1766364

[26] Lu J Wang W Mi Y Zhang C Ying H Wang L. AKAP95-mediated nuclear anchoring of PKA mediates cortisol-induced PTGS2 expression in human amnion fibroblasts. Sci Signal. (2017) 10:eaac6160. 10.1126/scisignal.aac6160 29162743

[27] Urieli-Shoval S Linke RP Matzner Y. Expression and function of serum amyloid A, a major acute-phase protein, in normal and disease states. Curr Opin Hematol. (2000) 7:64–9. 10.1097/00062752-200001000-00012 10608507

[28] Kovacevic A Hammer A Sundl M Pfister B Hrzenjak A Ray A. Expression of serum amyloid A transcripts in human trophoblast and fetal-derived trophoblast-like choriocarcinoma cells. FEBS Lett. (2006) 580:161–7. 10.1016/j.febslet.2005.11.067 16343490

[29] Sandri S Urban Borbely A Fernandes I de Oliveira EM Knebel FH Ruano R. Serum amyloid A in the placenta and its role in trophoblast invasion. PLoS ONE. (2014) 9:e90881. 10.1371/journal.pone.0090881 24614130 PMC3948705

[30] Cekmez Y Cekmez F Ozkaya E Pirgon O Yilmaz Z Yilmaz EA. Proadrenomedullin and serum amyloid a as a predictor of subclinical chorioamnionitis in preterm premature rupture of membranes. J Interferon Cytokine Res. (2013) 33:694–9. 10.1089/jir.2012.0134 24010826

[31] Ibrahim MI Ellaithy MI. Measurement of maternal serum amyloid a as a novel marker of preterm birth. J Matern Fetal Neonatal Med. (2019). 25:1–6. 10.1080/14767058.2019.1668370 31522581

[32] Yan H Zhu L Zhang Z Li H Li P Wang Y. HMGB1-RAGE signaling pathway in pPROM. Taiwan J Obstet Gynecol. (2018) 57:211–6. 10.1016/j.tjog.2018.02.008 29673663

[33] Menon R Richardson LS Lappas M. Fetal membrane architecture, aging and inflammation in pregnancy and parturition. Placenta. (2019) 79:40–5. 10.1016/j.placenta.2018.11.003 30454905 PMC7041999

[34] Lu Y Wang W Lin Y Lu J Li W Zhang C. Enhancement of cortisol-induced SAA1 transcription by SAA1 in the human amnion. J Mol Endocrinol. (2019) 62:149–58. 10.1530/JME-18-0263 30817315

[35] Challis JR Bloomfield FH Bocking AD Casciani V Chisaka H Connor K. Fetal signals and parturition. J Obstet Gynaecol Res. (2005) 31:492–9. 10.1111/j.1447-0756.2005.00342.x 16343248

[36] Wang W Chen ZJ Myatt L Sun K. 11beta-HSD1 in human fetal membranes as a potential therapeutic target for preterm birth. Endocr Rev. (2018) 39:241–60. 10.1210/er.2017-00188 29385440

[37] Kim YM Romero R Chaiworapongsa T Kim GJ Kim MR Kuivaniemi H. Toll-like receptor-2 and−4 in the chorioamniotic membranes in spontaneous labor at term and in preterm parturition that are associated with chorioamnionitis. Am J Obstet Gynecol. (2004) 191:1346–55. 10.1016/j.ajog.2004.07.009 15507964

[38] Elovitz MA Wang Z Chien EK Rychlik DF Phillippe M. A new model for inflammation-induced preterm birth: the role of platelet-activating factor and Toll-like receptor-4. Am J Pathol. (2003) 163:2103–11. 10.1016/S0002-9440(10)63567-5 14578208 PMC1892431

[39] Stampalija T Chaiworapongsa T Romero R Tarca AL Bhatti G Chiang PJ. Soluble ST2, a modulator of the inflammatory response, in preterm and term labor. J Matern Fetal Neonatal Med. (2014) 27:111–21. 10.3109/14767058.2013.806894 23688338 PMC4161022

[40] Okazaki T Casey ML Okita JR MacDonald PC Johnston JM. Initiation of human parturition. XII Biosynthesis and metabolism of prostaglandins in human fetal membranes and uterine decidua. Am J Obstet Gynecol. (1981) 139:373–81. 10.1016/0002-9378(81)90312-4 6781352

[41] Rehnstrom J Ishikawa M Fuchs F Fuchs AR. Stimulation of myometrial and decidual prostaglandin production by amniotic fluid from term, but not midtrimester pregnancies. Prostaglandins. (1983) 26:973–81. 10.1016/0090-6980(83)90158-2 6680924

[42] Cicarelli LM Perroni AG Zugaib M de Albuquerque PB Campa A. Maternal and cord blood levels of serum amyloid A, C-reactive protein, tumor necrosis factor-alpha, interleukin-1beta, and interleukin-8 during and after delivery. Mediators Inflamm. (2005) 2005:96–100. 10.1155/MI.2005.96 16030392 PMC1533909

