# Supplementary figures and images for: De novo Synthesis of SAA1 in the Placenta Participates in Parturition
Source: Front Immunol. 2020 Jun 9;11:1038. doi: 10.3389/fimmu.2020.01038 (PMC7297131; doi:10.3389/fimmu.2020.01038)

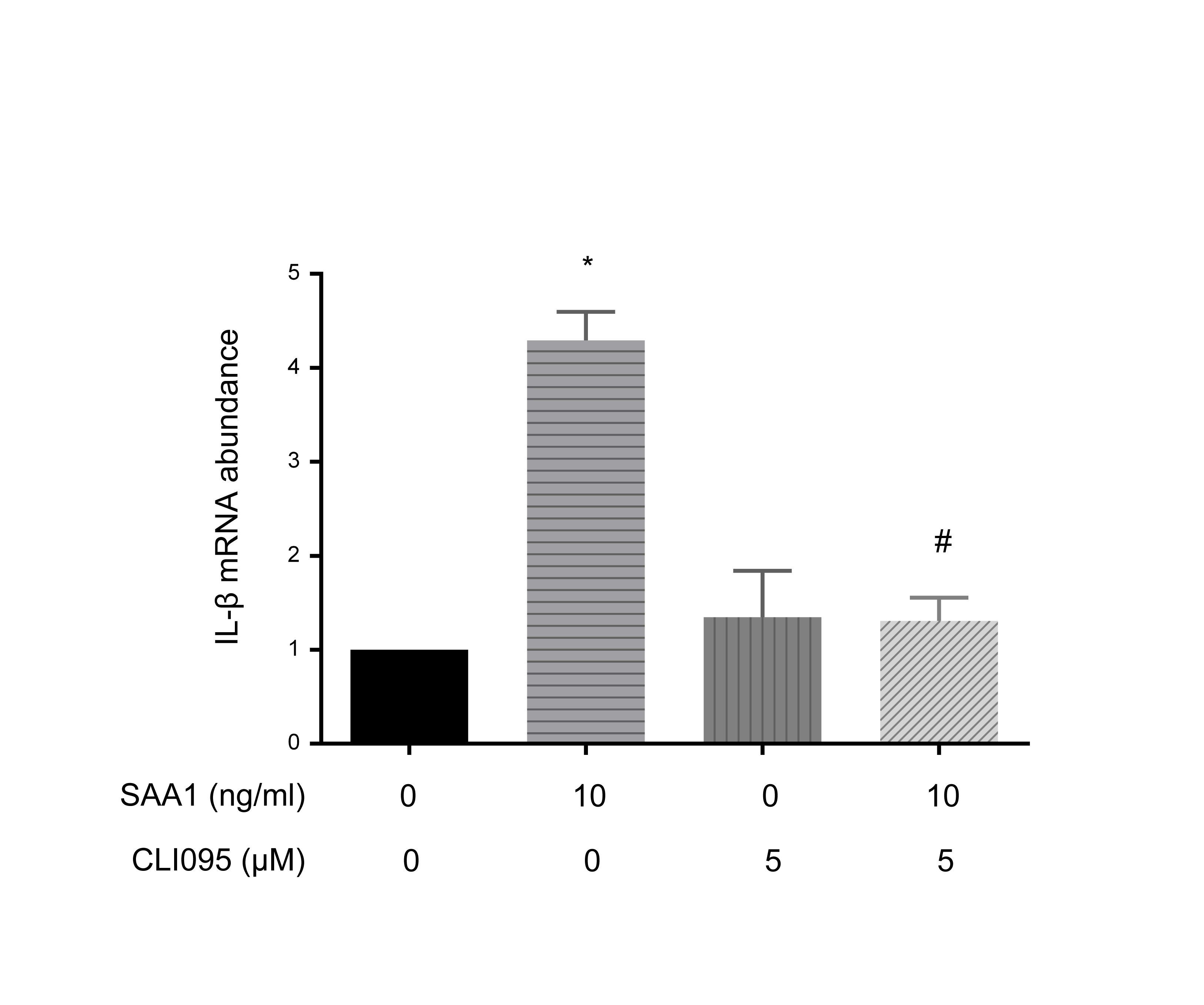

Supplement: Supplementary Figure 1 — Induction of IL-1β mRNA in human placental syncytiotrophoblasts by SAA1 (10 ng/mL, 24 h), which was blocked by CLI095 (5 μM, 24 h), a TLR4 inhibitor. n = 3. *P < 0.05 vs. control (0); #P < 0.05 vs. SAA1. [file Image_1.TIF]

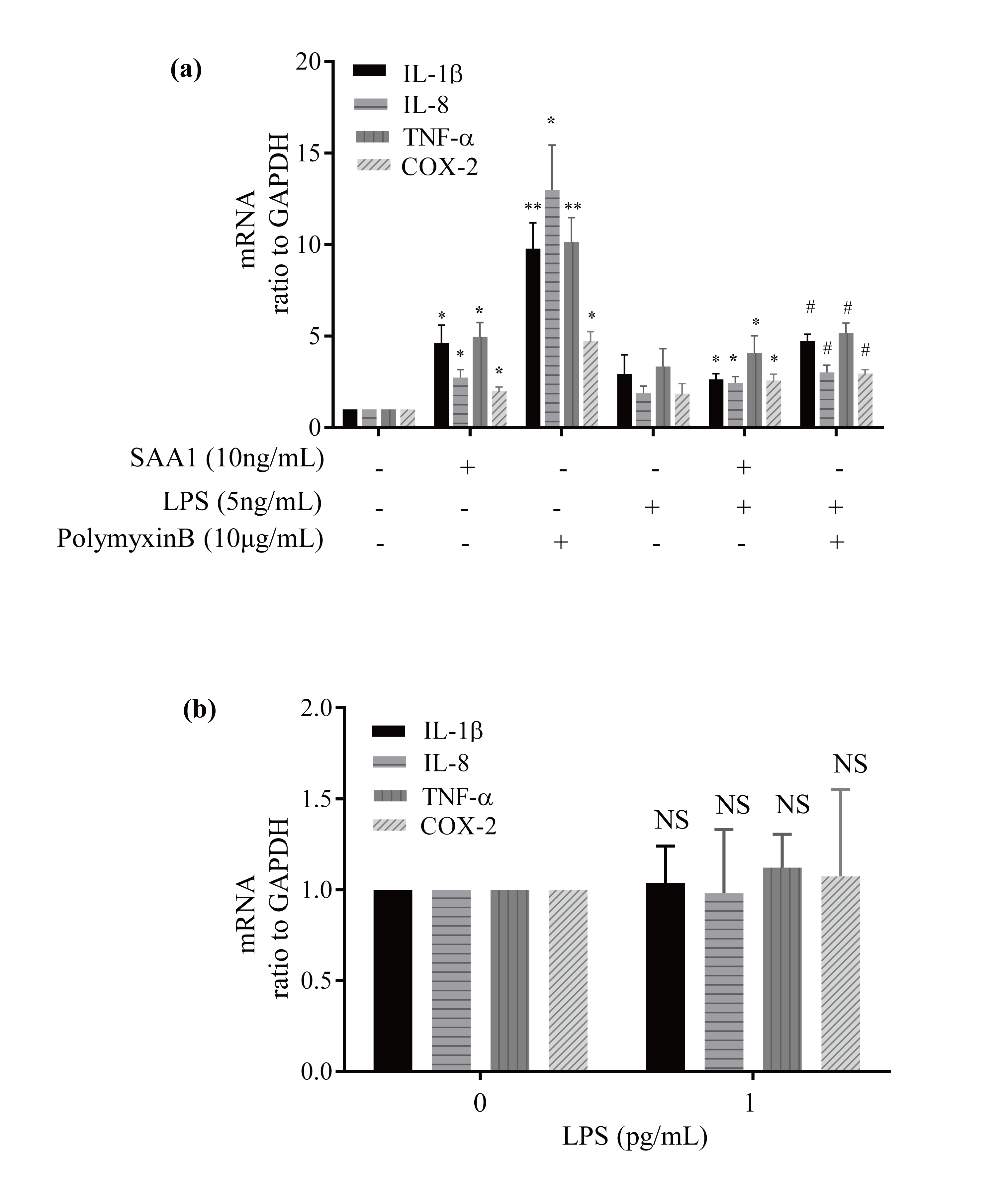

Supplement: Supplementary Figure 2 — Specificity examination of SAA1 effects in human syncytiotrophoblasts. (A) Polymyxin B (10 μg/mL, 24 h), an endotoxin inhibitor, had no effect on SAA1 (10 ng/mL)-induced changes in IL-1β, IL-8, TNF-α and COX-2 mRNA but blocked the effect of LPS (5 ng/mL) (n = 4). (B) Trace amount of LPS (1 pg/mL, 24 h), which is equivalent to the maximal amount contained in the recombinant SAA1, had no effect on the expression of IL-1β, IL-8, TNF-α, and COX-2 mRNA (n = 3). Data are mean ± SEM. *P < 0.05, **P < 0.01 vs. control (0); #P < 0.05 vs. LPS. [file Image_2.TIF]
